# Tweet you right back: Follower anxiety predicts leader anxiety in social media interactions during the SARS-CoV-2 pandemic

**DOI:** 10.1371/journal.pone.0279164

**Published:** 2023-02-09

**Authors:** Alexandros Psychogios, Dritjon Gruda, Adegboyega Ojo

**Affiliations:** 1 Birmingham City Business School, Birmingham City University, Birmingham, United Kingdom; 2 ALBA Graduate Business School, The American College of Greece, Agia Paraskevi, Greece; 3 National University of Ireland Maynooth, School of Business, Maynooth, Ireland; 4 School of Public Policy and Administration, Carleton University, Ottawa, Canada; 5 Department of Applied Informatics in Management, Gdansk University of Technology, Gdańsk, Poland; University of Albany, State University of New York, UNITED STATES

## Abstract

Recent research has shown that organizational leaders’ tweets can influence employee anxiety. In this study, we turn the table and examine whether the same can be said about followers’ tweets. Based on emotional contagion and a dataset of 108 leaders and 178 followers across 50 organizations, we infer and track state- and trait-anxiety scores of participants over 316 days, including pre- and post the onset of the SARS-CoV-2 pandemic and crisis. We show that although leaders traditionally possess greater authority and power than their followers, followers have the power to influence their leaders’ state anxiety. In addition, this influence is particularly strong in the case of less trait anxious leaders.

## Introduction

Leadership is a social phenomenon that requires at least two (or more) people who exchange information and coordinate actions to achieve specific common goals [1]. However, leadership also includes psychological and emotional adaptations of leadership agents, namely leaders and followers [2]. There is substantial evidence that demonstrates that leaders influence the emotional state of followers [3], a phenomenon called emotional contagion [4]. The reason is mainly linked to leaders’ status role, which corresponds to access to resources and authority [5]. Despite the hard evidence suggesting leaders’ effect followers’ emotional states, less is known about the effect of followers on leaders’ emotional states. Although there are few significant studies suggesting that followers seem to have an additional effect on their leaders’ emotional status [6], the question remains: do followers influence the emotional state of their leaders similarly to leaders influencing followers’ emotions? The present study attempts to fill this gap by presenting strong evidence suggesting that followers can influence their leaders’ emotions even via computer-mediated communication (CMC) on the social media platform Twitter.

### Emotional contagion: From unidirectional to bidirectional

Emotional contagion is the “tendency to automatically mimic and synchronize facial expressions, vocalizations, postures and movements with those of another person and, consequently, to converge emotionally” [7; p. 5]. Emotional contagion processes have been widely studied in organizational contexts, with previous studies finding that leaders’ moods can influence the mood of followers, impacting the performance of the latter [8, 9]. The transfer of emotional states from leaders to followers can also influence the perception of the latter towards the leader’s style, honesty, and overall effectiveness [10–13]. However, all of these studies have examined emotional contagion between leaders and followers as a unidirectional phenomenon, ignoring that the social interaction process is a process of mutual influence. This study builds on this argument and examined whether followers can influence leaders via emotional contagion in a particular setting, namely computer-mediated communication.

### Bidirectional anxiety contagion in computer-mediated communications

Within the realm of CMC, some studies have found that emotional contagion is not only possible in CMC but also that interactions on social media platforms allow the transfer of discrete emotions, including anger or happiness [14] as well as anxiety [15]. Social media platforms, especially public ones such as Twitter, allow the investigation of the effects of emotion transfer from followers to leaders and vice versa [16]. And indeed, less than a handful of studies have suggested that the transfer of emotions via social media platforms, in principle, can be a bi- or even multidirectional effect [14, 17, 18].

In the leadership literature, most attention is given to leaders’ effects on followers, though there is growing evidence that followers affect leaders in numerous ways and that the leadership process is indeed bi-directional. In the context of emotions, a similar pattern can be observed—with most attention given to the ways that leaders produce emotional effects on followers. Yet, this lopsided attention is incongruent with the fact that emotions are highly contagious, regardless of who is the ‘producer’ and observer of the emotions. Indeed, Kramer and colleagues [19] demonstrated that slight manipulations of the emotions ‘displayed’ by one’s friends resulted in the observers displaying similar emotions. Hence, if reading about another person’s emotions (and indeed, these may be people the observer hasn’t spoken to in years) on a screen creates evidence of emotional contagion, it is reasonable to believe that leaders and followers, who typically interact frequently, may be prone to similar emotional contagion in both leader-follower and follower-leader directions.” However, conclusive empirical evidence to that effect remains elusive. In this respect, this study both builds on existing literature and expands prior empirical evidence by investigating the transfer of a particular emotion, namely anxiety, within a CMC context.

Anxiety is present in both state and trait forms [20]. And while state anxiety is short-term and event-specific (e.g. anxiety due to pandemic effects, etc.), trait anxiety refers is a stable trait-like form of anxiety, similar to other personality traits [20, 21]. In terms of behavior, individuals who score high on trait anxiety tend to be both more anxious than others (i.e., intensity) and experience anxiety more often (i.e., frequency).

We argue that state anxiety is a particularly important emotion to study given the increased likelihood of negative emotions spreading more easily on social media than positive emotions. In addition, while we recognize that individual responses in the context of the SARS-CoV-2 crisis individuals vary [22], anxiety is considered the default crisis emotion [23] and is experienced by all individuals during a crisis before they experience any of the other crisis-related emotions (i.e., fear, anger, sadness). Therefore, we argue that the study of state anxiety is particularly important [24] when assessing communications between followers and leaders in a social media context. And while several previous studies have found that leaders’ state anxiety influences followers’ state anxiety [25, 26], including in social media contexts (e.g., [20]), to the best of our knowledge, there are no previous studies to examine anxiety contagion from followers and leaders on social media. Yet, based on previous evidence in face-to-face teams [6] we expect that follower state anxiety in CMC influences leader state anxiety over time.

*H1*: *In CMC*, *follower state anxiety positively predicts leader state anxiety*.

In addition, trait anxiety, in combination with state anxiety, can influence the way that a person experiences anxiety in a specific context [24]. Previous studies have also shown that the role of leader trait anxiety in particular is an important moderator to consider in the transfer of anxiety from leaders to followers [15]. We argue that leader trait anxiety also forms an important variable in the presented study. That is because highly trait anxious leaders (and individuals more broadly) are more anxious in general. Individuals who are pre-disposed to experience trait anxiety tend to perceive given situations as more hostile, and therefore give more attention to negative information [27]. And because such individuals are likely to be constantly on the alert and the lookout for possible threats, we would expect highly trait anxious leaders to be less influenced by their followers’ emotions, because they are predisposed to being anxious already. Put differently, we would expect the effect of followers’ state anxiety to be most impactful on less trait anxious leaders’ state anxiety. Hence, we would expect leaders with higher trait anxiety to be less influenced by their followers than less trait anxious leaders.

*H2*: *In CMC*, *leader trait anxiety moderates the relationship between follower state anxiety and leader state anxiety*, *so that increased follower state anxiety is more likely to predict increased leader state anxiety in the case of less trait anxious leaders*.

## Methodology

The presented analyses and results are based on a publicly accessible dataset composed by Gruda, Ojo and Psychogios [15]. The original dataset features 43,283 matched daily leader-follower observations of 197 leaders and 958 of their followers across 79 companies engaging in CMC on the public social media platform Twitter. Gruda, Ojo and Psychogios [15] examined the influence of leaders’ state anxiety on follower state anxiety but ignored examining their data for a possible bidirectional effect of anxiety contagion from followers to leaders. However, the provided public dataset includes all the required data to test for such an effect (except follower status, based on job title, which were obtained directly from the authors of the original study). Hence, we base the presented study on this dataset to conduct our analyses. We provide a brief overview of the measures of the various variables in this paper, while a more detailed overview of the various measures can be found in the original manuscript.

### Sample and procedure

The final sample consisted of both the leaders and followers. Leaders were defined as top managers, namely C-suite executives, i.e., individuals with a job title of e.g., Chief Executive Officer (or CEO), Chief Financial Officer (or CFO), etc. However, unlike Gruda and Ojo [15], we did not classify all remaining individuals in the provided dataset as followers. We argue that it is unlikely that lower-level followers (e.g., analysts) can influence top-management leaders in the same manner that top leaders can influence their employees. That is because C-suite executives are considered senior organizational leaders [5] who wield great signaling power due to their access to privileged information, access to resources, and resource distribution power [28]. This power is not available to all other followers. However, we would expect that direct followers of such leaders or followers who are close to top leaders in the organizational hierarchy (e.g., Directors, Senior Vice Presidents, Head of Departments, etc.) do have the power to influence their leaders and affect their emotions. Hence, we specified such individuals as followers. Follower status scores were obtained from the original authors [20]. All employees were from the United States of America. This is because the ground truth training data was obtained exclusively from individuals in the United States. This led to a final dataset of 108 leaders and 178 followers across 50 organizations and a total of 6,988 dyadic daily interaction observations.

### Measures

#### State and trait anxiety

State and trait anxiety scores were determined based on the anxiety detection algorithm described by Gruda and Hasan [29]. As stated in Gruda, Ojo and Psychogios [15] “this algorithm was trained on a dataset of 600 randomly selected tweets from 10,386 users, scored by 604 zero-acquaintance human raters from the United States based on a six-item short-form of the Spielberger State-Trait Anxiety Inventory [STAI; 29]. On average, each tweet was rated five times”, on a scale from 1 (“Not at all”; low anxiety) and 4 (“Very much”; high anxiety) by each rater. The training procedure involved the use of a 6-fold cross-validation resampling plan and resulted in a model with R^2^ = 0.49 and a Root-Mean-Square Error (RMSE) of 0.52, and was validated using a set of 3.33 million tweets. Trait anxiety was determined as a 30-day average of state anxiety scores per user before the examined time period (before 5^th^ October 2019). More information about the algorithm is provided in Gruda and Hasan [30] and Gruda and Hasan [31].

#### Big-five personality traits

We also included the Big Five personality traits as controls in our analysis. Previous work [e.g., 32–35] has demonstrated that social media data provides a continuous stream of data that can be used to accurately and reliably measure personality traits. The Big Five personality traits for all individuals in our sample were obtained using the IBM Watson Personality Insights API [36], an open-vocabulary machine-learning approach trained using a reference sample of 1,000,000 individuals. The IBM Watson algorithm also provides six individual facets for each Big Five personality dimension [21, 37], which were combined into higher-order Big Five personality dimensions (all Cronbach α ≥ .70, except for Openness to Experience: α = .68, see Table 1).

**Table 1:** Pairwise correlations of main variables.

			M	SD	1	2	3	4	5	6	7	8	9
Leader	1	State Anxiety (Day 2)	2.13	0.17	-								
	2	State Anxiety (Day 1)	2.10	0.16	0.62***	-							
	3	State Anxiety (Day 0)	2.10	0.17	0.58***	0.56***	-						
	4	Trait Anxiety	2.12	0.10	0.58***	0.56***	0.58***	-					
	5	Openness to Experience	0.62	0.10	-0.04***	0.14	0.07	0.26**	(.68)				
	6	Conscientiousness	0.67	0.13	-0.35***	-0.32***	-0.36***	-0.67***	-0.25**	(.86)			
	7	Extraversion	0.59	0.17	-0.44***	-0.42***	-0.45***	-0.73***	-0.20*	0.76***	(.90)		
	8	Agreeableness	0.51	0.12	-0.41***	-0.28**	-0.36***	-0.55***	0.23*	0.71***	0.77***	(.73)	
	9	Neuroticism	0.27	0.19	0.35***	0.37***	0.36***	0.69***	0.28*	-0.94***	-0.84***	-0.72***	(.95)
	10	Gender	0.85	0.36	0.14	-0.04	-0.01	-0.00	-0.18	0.00	-0.17	-0.16	0.02
Follower	1	-	-	-	-								
	2	State Anxiety (Day 1)	2.21	0.28	-	-							
	3	State Anxiety (Day 0)	2.21	0.28	-	0.66***	-						
	4	Trait Anxiety	2.20	0.11	-	0.53***	0.58***	-					
	5	Openness to Experience	0.40	0.10	-	0.19*	0.18*	0.33***	(.68)				
	6	Conscientiousness	0.54	0.14	-	-0.35***	-0.36***	-0.65***	-0.35***	(.87)			
	7	Extraversion	0.42	0.16	-	-0.49***	-0.44***	-0.72***	-0.18*	0.74***	(.90)		
	8	Agreeableness	0.56	0.13	-	-0.37***	-0.26***	-0.52***	0.18*	0.62***	0.68***	(.72)	
	9	Neuroticism	0.44	0.19	-	0.38***	0.40***	0.69***	0.42***	-0.93***	-0.80***	-0.56***	(.95)
	10	Gender	0.74	0.44	-	0.20*	0.15*	0.20**	-0.16*	-0.14	-0.31***	-0.42***	0.12

Note: Original Cronbach’s alphas as outlined in Gruda et al. (2022) [20] in parentheses; Gender coded as 0 (female) and 1 (male);

*** p < .001,

** p < .01,

* p < .05;

n = 108 leaders, 178 followers.

#### Post-annotation dataset consolidation

State and trait anxiety scores were merged with the predicted Big Five personality traits for both leaders and followers and a pairwise observation dataset based on the day of the respective tweet was created. For example, tweets by followers from 1^st^ March 2020 were paired with leader tweets of the same day. The provided data spanned from 5^th^ October 2019 to 13^th^ August 2020, including 158 days before the onset (i.e., onset is 11^th^ March 2020) and 158 days since the onset of the SARS-CoV-2 pandemic.

### Analytical strategy

As followers are nested within leader-follower dyads (i.e., several followers per organizational leader) and within companies, we use a multilevel cross-classified mixed-effects model for repeated measures to test our hypotheses [38]. We define a three-level model with random intercepts at the company level, using the Aikake Information Criterion (AIC) and the Bayesian Information Criterion (BIC) to compare models. All analyses were conducted using Stata 17.0.

## Results

Pairwise correlations on the leader and follower level and summary statistics are provided in Table 1. We should note that the correlations among our Big Five measures may, at first, appear quite high. However, it is important to remember that our personality scores were derived based on inferences from textual data. Hence, we did not ask participants directly to complete traditional self-report (i.e., direct) personality measures. Put differently, the displayed traits are indeed only expressed traits, not actual traits. As such, the difference between inferred and direct measurement partially explains why intercorrelations between inferred traits tend to be higher (e.g., r >.70) compared to the traditional assessment of self-report scales [38].

We run a similar model as outlined in Gruda, Ojo and Psychogios [15], however, in this study, we now investigate the effect of follower state anxiety (Day 0) on leader state anxiety (Day 2). We apply this multi-day lagged regression design to account for the effect of a potential third variable [17] and potential sleeper effects of the follower-leader anxiety influence. Notably, unlike Gruda, Ojo and Psychogios [15], we specify time as a continuous (not categorical) moderator in our model. This allows us to consider the full dataset and minimize the loss of any variance between the obtained anxiety scores over time. Results of the specified multi-level cross-classified mixed-effects model for repeated measures are shown in Table 2.

**Table 2:** Regression interaction between follower state anxiety (Day 0) and leader trait anxiety on leader state anxiety (Day 2).

	*b*	*SE*	*z*	*95% CIs*
Time	-0.008	0.005	-1.63	[-.0197; 0.002]
Follower State Anxiety (Day 0)	0.064	0.217	0.30	[-0.360; 0.489]
Leader Trait Anxiety	0.826***	0.245	3.39	0.345; 1.304
Time X Follower State Anxiety (Day 0)	0.005*	0.002	2.06	[0.000; 0.010]
Time X Leader Trait Anxiety	0.004	0.003	1.62	[-0.001; 0.009]
Follower State Anxiety (Day 0) X Leader Trait Anxiety	-0.023	0.102	-0.22	[-0.222; 0.177]
Time X Follower State Anxiety (Day 0) X Leader Trait Anxiety	-0.002*	0.001	-2.01	[-0.005; -0.001]
Leader State Anxiety (Day 0)	0.047***	0.012	3.93	[0.023; 0.070]
Leader State Anxiety (Day 1)	0.051***	0.012	4.24	[0.027; 0.074]
Follower State Anxiety (Day 1)	0.023*	0.012	1.99	[0.000; 0.046]
Follower Trait Anxiety	-0.078	0.065	-1.2	[-0.206; 0.050]

Note: The presented model includes several controls, including leader and follower Big Five personality traits, leader and follower gender, and organization size (categorical);

*** *p* < 0.001,

** *p* < 0.01,

* *p* < 0.05;

n = 6,988 observations.

**Fig 1:**
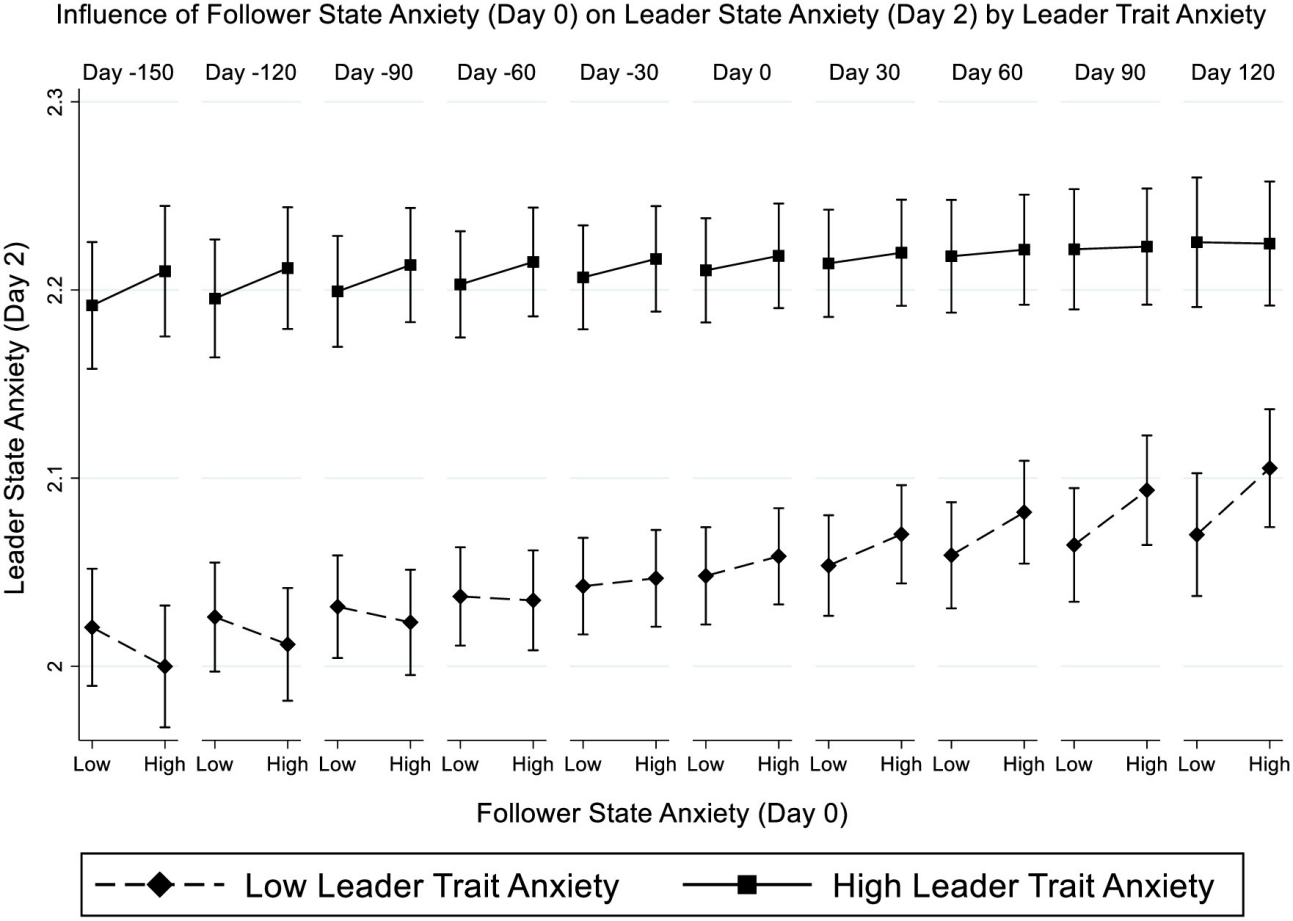
Regression interaction between follower state- and leader trait anxiety on leader state anxiety over time.

All outlined models in Table 2 include various control variables, including the Big Five personality traits and follower and leader state anxiety on the preceding days (i.e., Day 0) and leader state anxiety on the preceding days (i.e., Day 0 and Day 1). We find a significant three-way interaction, between leader state- and trait-anxiety on follower anxiety and time (Table 2: *b* = -0.002, *SE* = 0.001, *z* = -2.01, *p* = 0.044). To better understand this interaction, we plotted the results of the complete model (Table 2) in Fig 1.

Graphing this three-way interaction (+/- 1 SD, Fig 1) showed that in the case of less trait anxious leaders, follower state anxiety on Day 0 positively predicted leader state anxiety two days later (i.e., Day 2), however, only in the case of less trait anxious leaders. In addition, the effect was only present after the onset of the SARS-CoV-2 pandemic and increased as the crisis continued (e.g., Day 60: simple slope = 0.04, *SE* = 0.02, *z* = 1.96, *p* = .05; Day 90: simple slope = 0.05, *SE* = 0.02, *z* = 2.11, *p* = .035; Day 120: simple slope = 0.06, *SE* = 0.03, *z* = 2.17, *p* = .030). These results indicate that the context in which the CMC occurs matters greatly for emotional contagion between followers and leaders.

### Robustness check

Anxiety is closely related to neuroticism, as is also evident in this study (see Table 1). To check whether results diverge when considering alternative models which do not include neuroticism as a control, we rerun a separate model without this control. Results were comparable and remained robust (*b* = -.002, *t* = -2.01, *p* = 0.045).

## Discussion and implications

Given that there is a lack of knowledge regarding emotional contagion in CMC, this study investigates anxiety contagion from followers to leaders when interacting on Twitter. Keeping in mind the findings by Gruda, Ojo and Psychogios (2022), the presented study suggests that the anxiety contagion process in follower-leader interactions is bidirectional. We have known for some time that a unidirectional contagion exists, namely in that leaders’ emotional state influences followers, sometimes via a spread of emotional contagion from leader to followers to other followers [39]. In addition, a previous recent study also showed that a leader’s state anxiety influences followers’ state anxiety even via interactions on social media [15]. In the present study, for the first time, we focused on investigating the opposite direction of emotional influence, namely from followers to leaders within a social media context.

This study shows that in a CMC interaction follower state anxiety predicts leader state anxiety, even when accounting for a series of leader and follower personality traits and demographics. In addition, this study found that leaders’ trait anxiety moderates the relationship between follower state anxiety and leader state anxiety. In other words, increased followers’ state anxiety results in increased levels of leaders’ state anxiety especially when the latter demonstrate less trait anxiety levels. The results above seem to support similar studies that found that followers’ emotional states influence leaders’ emotions and in turn leaders’ performance [3, 6]. In other words, this study provides strong empirical evidence supporting the argument that followers are not powerless and can indeed influence their leaders’ emotional states even when interacting online.

Finally, another contribution of this study is showing that emotional contagion can also happen through communication on social media. It seems that social media have become a critical factor in influencing behavior in organizations [40]. Tweeting messages to each other seems to matter in terms of both anxiety expression [20, 21] and anxiety contagion [15, 37]. This implies that leaders should carefully consider the messages they communicate via social media to followers, not only because there are good chances to transfer anxiety to the latter, but also because the latter can ‘hit’ back with equally stressful messages which influence their leaders in turn. In our study, we find that this, indeed, seems to be the case, particularly in a crisis context like the recent SARS-CoV-2 pandemic, during which leaders had to take action and respond (through social media as well) to followers’ affect and demands [41]. Leaders and followers need to understand that this can have a negative impact not only on each other’s mental health but also can harm their own well-being [42].

### Limitations and future research

Our study has some limitations. Firstly, because our data relies on follower and leader tweets, it could be that social media users do not present their own selves but rather a positively enhanced and promotable version of themselves when communicating online. While this argument makes intuitive sense, previous research has found strong support for negating this belief [34, 43]. In addition, we would argue that the applied methodology is particularly suited to detect anxiety in an unobtrusive manner [31]. Put differently, due to behavioral residue found in tweets, the applied approach allows us to detect changes in anxiety more accurately [21].

Secondly, our data was solely restricted to the U.S., due to the training dataset of the applied algorithm. However, because of the country’s size, the infection rate of SARS-CoV-2 differed in time from state to state and indeed even from county to county. Hence, a more detailed assessment of COVID-19 infections over time could have provided different results concerning the impact of the crisis on follower anxiety. We encourage future research to examine this more closely.

Finally, another important limitation is the issue of endogeneity and lack of causality in the presented model. To prove causality, a randomized control trial would be needed, in which individuals (leader and followers) would be randomly assigned into two groups, and followers would be asked to tweet highly anxious or less anxious tweets. Leaders, themselves divided into two groups (i.e., highly trait anxious vs low trait anxious) in turn, would be asked to read these tweets and provide self-report ratings of their respective state anxiety at that moment. In addition, we also were interested in testing the lagged influence of follower state anxiety and leader state anxiety. Therefore, after the experiment takes place, we would also need to test leader state anxiety several days later. However, such an experimental setup—albeit it would allow researchers to visualize the direction and measurement of the contagion effect—would prove difficult to implement and would lack generalizability, due to the artificial conditions of the aforementioned laboratory experiment. We argue that the presented approach using machine learning to infer (state and trait) anxiety of both leaders and followers, allowed us to test the contagion effect in a naturalistic and unobtrusive setting. Finally, the applied multi-day lagged design allowed us for associations between follower and leader anxiety over time.

## Conclusion

Using a longitudinal research design with daily dyadic observations over 316 days—covering pre- and post-onset of the SARS-CoV-2 pandemic—and a machine learning approach, we examine anxiety contagion in CMC from followers to leaders. We found that follower state anxiety predicts leader state anxiety over time, while leader trait anxiety acts as an important moderator.
